# ITF promotes migration of intestinal epithelial cells through crosstalk between the ERK and JAK/STAT3 pathways

**DOI:** 10.1038/srep33014

**Published:** 2016-09-12

**Authors:** Juan Le, Duan Y. Zhang, Yong Zhao, Wei Qiu, Peng Wang, Yong Sun

**Affiliations:** 1 Department of Burn Surgery, Huaihai Hospital affiliated to Xuzhou Medical College, Xuzhou, Jiangsu Province, China; 2 Department of Burn Surgery, No. 97 Hospital of PLA, Xuzhou, Jiangsu Province, China

## Abstract

Intestinal trefoil factor (ITF), a member of the trefoil factor family, is a “Super-protective factor” for intestinal mucosal protection. This study was designed to explore the mechanism by which ITF promotes intestinal epithelial cell migration. Intestinal epithelial cells were treated with the human ITF (hITF). Phospho-ERK, phospho-STAT3 Tyr^705^, and phospho-STAT3 Ser^727^ levels were detected at different time points by western blot. To assess the potential crosstalk between the ERK and JAK/STAT3 pathways, HT-29 cells were treated with the MEK-inhibitor, U0126, and phosphor-STAT3 levels were evaluated. Conversely, cells were treated with the JAK-inhibitor, AG490, and ERK-activity was evaluated. Transwell assay was performed to investigate the effect of the crosstalk on the cell motility. *MMP-2* and *MMP-9* transcription was analyzed by quantitative real-time PCR. E-cadherin degradation was detected by immunofluorescence. Our results indicate that hITF simultaneously activated the ERK and JAK/STAT3 pathways and a crosstalk was detected between the two pathways. hITF increased cell migration. This effect was abolished by U0126 and AG490 treatment. hITF increased *MMP2* and *MMP9* mRNA levels and E-cadherin degradation and U0126 and AG490 abolished this effect of hITF. In conclusion, the hITF-induced crosstalk between the ERK and JAK/STAT3 pathways is associated with intestinal epithelial cell migration.

The intestinal mucosal barrier possesses multiple functions. It consists of a mechanical barrier, a mucosal barrier, a microbial barrier, and an immune barrier. Serving as the center of traumatic stress, the intestinal mucosa is vulnerable to anoxia, ischemia, severe trauma, and generalized infection^1^. Therefore, protection of the intestinal mucosa barrier and restoration of impaired intestinal mucosa are hot topics of basic research and clinical therapy, which urgently need to be clarified.

The intestinal trefoil factor (ITF) is a low-molecular weight polypeptide reported to protect and repair the gastrointestinal mucosa through the maintenance of intestinal epithelial cell integrity and restoration of normal intestinal permeability^2^. ITF has a unique domain in which six cysteine residues in a sequence of 38 or 39 amino acid residues form three disulfide bonds. And ITF/TFF3 homodimer has seven cysteine residues participating in disulphide bonds- the seventh links the two subunits. This unique structure makes it resists degradation by proteolytic enzymes and extreme pH, so that it can exert its physiological functions in the gastrointestinal tract^3^. As an essential regulatory protein of mucosal reconstruction, ITF plays an important role in the protection and restoration of the intestinal mucosa. However, its mechanism of action remains unclear.

ITF promotes cell migration of impaired intestinal mucosa through phosphorylation and activation of ERK1/2^4^. However, blocking the ERK signaling pathway did not fully suppress ITF-induced cell migration, suggesting that other signaling pathways are involved. Our previous study demonstrated that ITF can activate the JAK-STAT3 signaling pathway and, thus, promote its self-transcription (unpublished). The crosstalk between the ERK and JAK/STAT3 pathways has been confirmed in related studies^5^,^6^. Therefore, we hypothesized that ITF may facilitate intestinal mucosal reconstruction via the crosstalk between the ERK and JAK-STAT3 pathways.

In this study, we employed a human intestinal epithelial system, in which HT-29 cell line was cultured*in vitro*. Treatment of HT-29 cells with the ERK inhibitor, U0126, or with the JAK inhibitor, AG490, and evaluation of phosphoSTAT3-level or ERK-activity, respectively, provided information about the crosstalk between ERK and JAK/STAT3 pathways. We performed transwell assays to assess the migratory ability of HT-29 cells after treatment with hITF alone or in the presence of ERK and JAK inhibitors. *MMP2* and *MMP9* transcription and E-cadherin degradation were analyzed to identify the downstream targets of ITF promoting cell migration. Our aim was to elucidate the interactions between the ERK and JAK/STAT3 signaling pathways in regulating human intestinal epithelial cell migration promoted by ITF and to lay the foundation for the protection of the intestinal mucosa.

## Results

### hITF production

**Figure 1:**
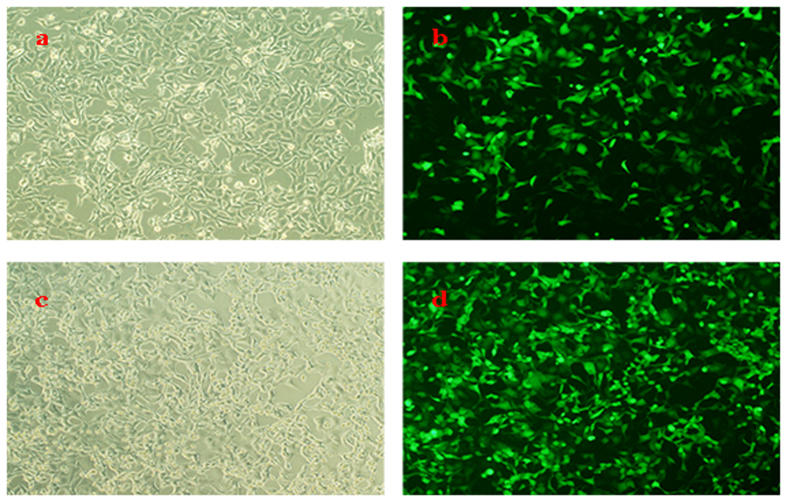
hITF production. (**a**) HEK293 cells infected with the recombinant adenovirus containing the human ITF gene (Ad-hITF) for 20 h were observed under white light. (**b**) HEK293 cells observed under a fluorescence microscope after 20 h (**c**) HEK293 cells infected with the recombinant human ITF adenovirus for 44 h were observed under white light. (**d**) HEK293 cells observed under a fluorescence microscope after 44 h. The scale bar in insets represents 50 μm (original magnification: 40×).

HEK293 cells were infected with Ad-hITF in order to produce hITF (Fig. 1). HT-29 cells were treated with hITF at a concentration of 60 μg/mL hITF in the following experiments.

### hITF activates the Ras/MAPK and JAK/STAT3 pathways in HT-29 cells

**Figure 2:**
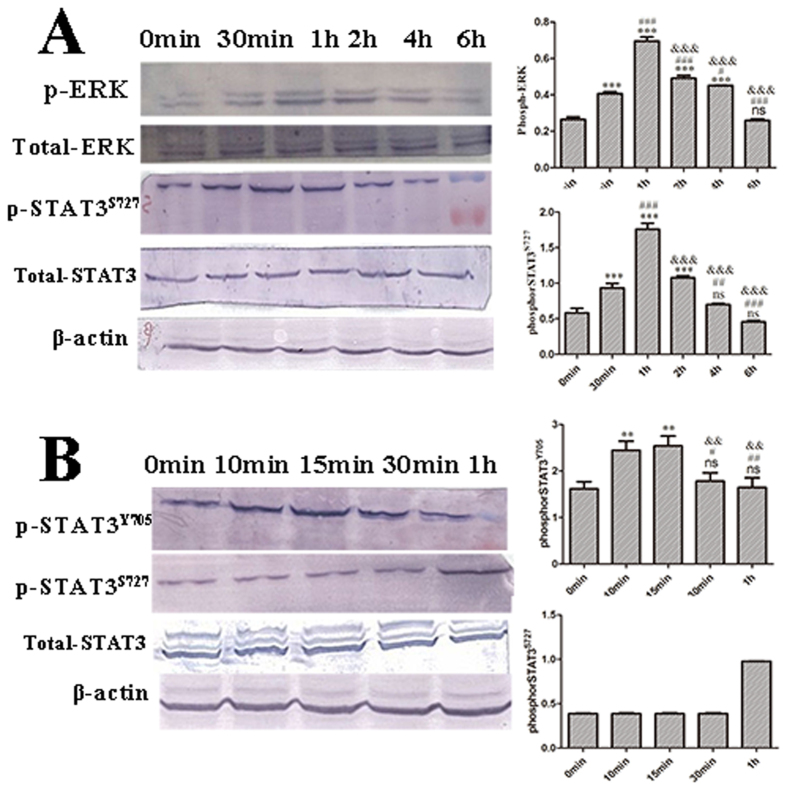
Effect of hITF on phospho-ERK, phospho-STAT3^S727^ and phospho-STAT3y705 levels. (**A**) Effect of hITF on phospho-ERK and phospho-STAT3^S727^ levels. HT-29 cells were treated with 60 μg/mL of hITF and the cell proteins were collected after 30 min, 1 h, 2 h, 4 h, and 6 h of stimulation (*P < 0.05, **P < 0.01, ***P < 0.001, ns no significant difference vs. 0 min; ^#^*P* < 0.05, ^##^*P* < 0.01, ^###^*P* < 0.001 vs. 30 min; ^&&&^*P* < 0.001 vs. 1 h).(**B**) Effect of hITF phospho-STAT3^y705^ and phospho-STAT3^S727^ levels. The cells were lysed at 10 min, 15 min, 30 min, and 1 h after treatment with the indicated hITF concentrations. Each value is the mean ± SD of three independent experiments with triplicate measurement (**P < 0.01, ***P < 0.001, ns no significant difference vs. 0 min; ^#^*P* < 0.05, ^##^*P* < 0.01, vs. 10 min; ^&&^*P* < 0.001 vs. 15 min).

In order to study the stimulatory effect of hITF on the Ras/MAPK and JAK/STAT3 signaling pathways in epithelial human intestinal epithelial cells, a time course experiment with hITF at the concentration of 60 μg/mL was performed using HT-29 cells. After hITF stimulation, the level of phospho-ERK1/2 was increased in a time-dependent manner and peaked at 1 hour with a 2-fold increase compared to the control group (Fig. 2A). Similarly, the level of phospho-STAT3 Tyr^705^ peaked at 15 min after stimulation with 60 μg/mL hITF with a 1-fold increase compared to the control group (Fig. 2B). Nevertheless, phospho-STAT3 Ser^727^ levels peaked later, mostly at 1 hour after stimulation, similar to the peak time of ERK (Fig. 2A,B). No change in total ERK and STAT3 protein levels was observed.

### Crosstalk between the ERK and JAK/STAT3 pathways

We demonstrated the activation of both pathways in the presence of hITF. We then analyzed the interaction between the ERK and JAK/STAT3 pathways. Treatment with the MEK-inhibitor, U0126, or the JAK inhibitor, AG490, and evaluation of phosphor-STAT3 levels or ERK activity, respectively, provided information about the crosstalk between the ERK- and JAK/STAT3 pathway.

**Figure 3:**
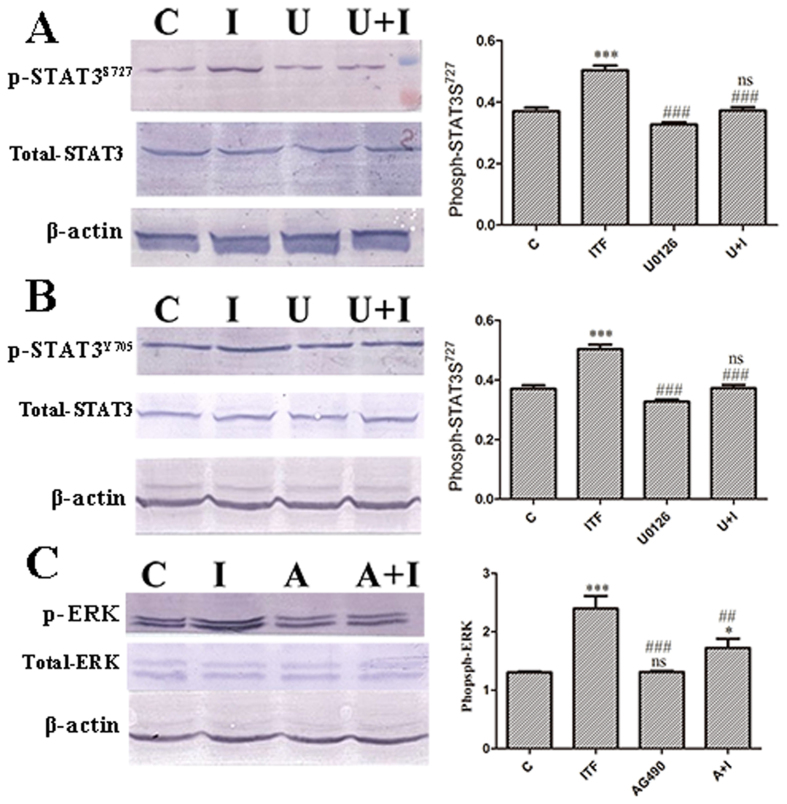
Effect of hITF on the crosstalk between the ERK and JAK/STAT3 pathways. (**A**) Effect of MEK inhibitor (U0126) on phospho-STAT3^S727^ levels. HT-29 cells were pretreated with U0126 for 2 h and then treated with hITF. Proteins were extracted at 1 h after treatment (***P < 0.001, ns no significant difference vs. control group; ^###^*P* < 0.001 vs. 30 min ITF group). (**B**) Effect of MEK inhibitor on phospho-STAT3y705 levels. Proteins were extracted at 15 min after treatment (***P < 0.001, ns no significant difference vs. control group; ^###^*P* < 0.001 vs. 30 min ITF group). (**C**) Effect of the JAK inhibitor (AG490) on phospho-ERK. HT-29 cells were pretreated with AG490 for 3 h prior to treatment with hITF (*P < 0.05, ***P < 0.001, ns no significant difference vs. control group; ^##^*P* < 0.01, ^###^*P* < 0.001 vs. ITF group). Proteins were collected at 1 h after treatment. Bonferroni’s multiple comparison post-hoc tests after one-way analysis of variance were used to assess statistical significance.

ERK inhibition resulted in a 1 time decrease in phosphorylation levels of STAT3 Ser^727^ (Fig. 3A) compared with that of the hITF group, but no significant change in phospho-STAT3 Tyr^705^ levels was observed (Fig. 3B). Conversely, treatment of HT-29 cells with the JAK inhibitor AG490 (A) alone or followed by treatment with ITF resulted in a 1 time decrease in ERK phosphorylation compared to the hITF-treated group (Fig. 3C).

### Effect of the ERK-JAK/STAT3 crosstalk mechanism on hITF-induced cell migration

**Figure 4:**
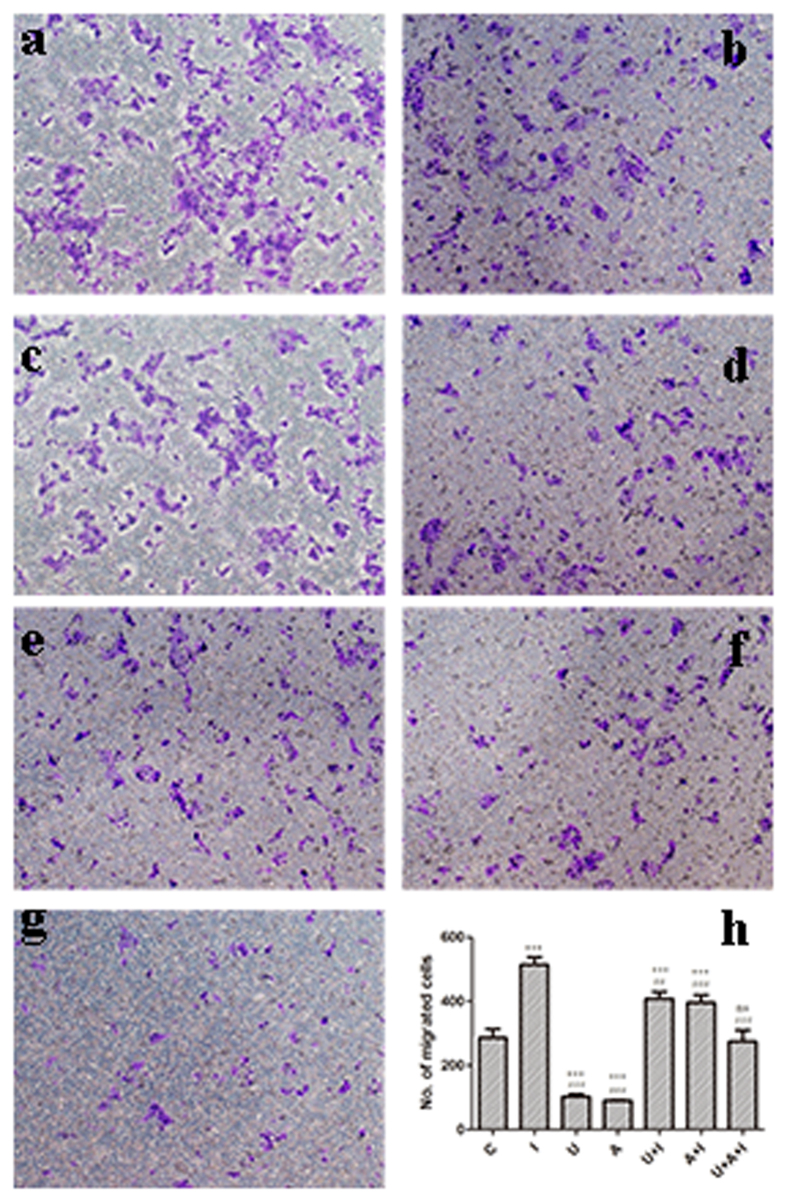
Effect of the crosstalk mechanism on hITF-induced cell migration. Cells were seeded in a transwell chamber and allowed to migrate across the chamber toward cell-specific conditioned medium for 48 h. Photomicrographs of stained migrating cells were taken under bright field illumination. Representative images are shown for HT-29 control cells (**a**), HT-29 cells treated with hITF (**b**), treatment with U0126 (**c**) and AG490 (**d**), pretreatment with U0126 (**e**) and AG490 (**f**) followed by exposure to hITF, pretreatment with both U0126 and AG490 followed by exposure to hITF (**g**). Migration is expressed as the number of migrated cells in five random microscopic fields per well (Mean ± SD; ***P < 0.001 ns no significant difference vs. control group; ^###^*P* < 0.001 vs. ITF group) (**h**). Results were obtained from three separate experiments. The scale bar in insets represents 50 μm (original magnification: 40×).

In order to investigate the effect of the ERK-JAK/STAT3 crosstalk mechanism on cell migration, a transwell assay was performed. HT-29 cells were pretreated with U0126 and AG490 and then exposed to hITF for 24 h. hITF strongly stimulated the migration of HT-29 cells through the matrigel by 179.7% compared to that of the vehicle-treated control cells. Pretreatment of HT-29 cells with U0126 or AG490, inhibited the hITF-induced cell migration to 77.1–79.4% compared to that of the hITF-treated group. Pretreatment with U0126 and AG490 in combination resulted in a decrease of hITF induced migration of HT-29 cells to 53.3% compared to that of the hITF-treated group, which was similar to the migration of vehicle-treated control cells (Fig. 4).

### ITF-induced cell migration is associated with an increase expression of matrix metalloproteinases (MMPs)

**Figure 5:**
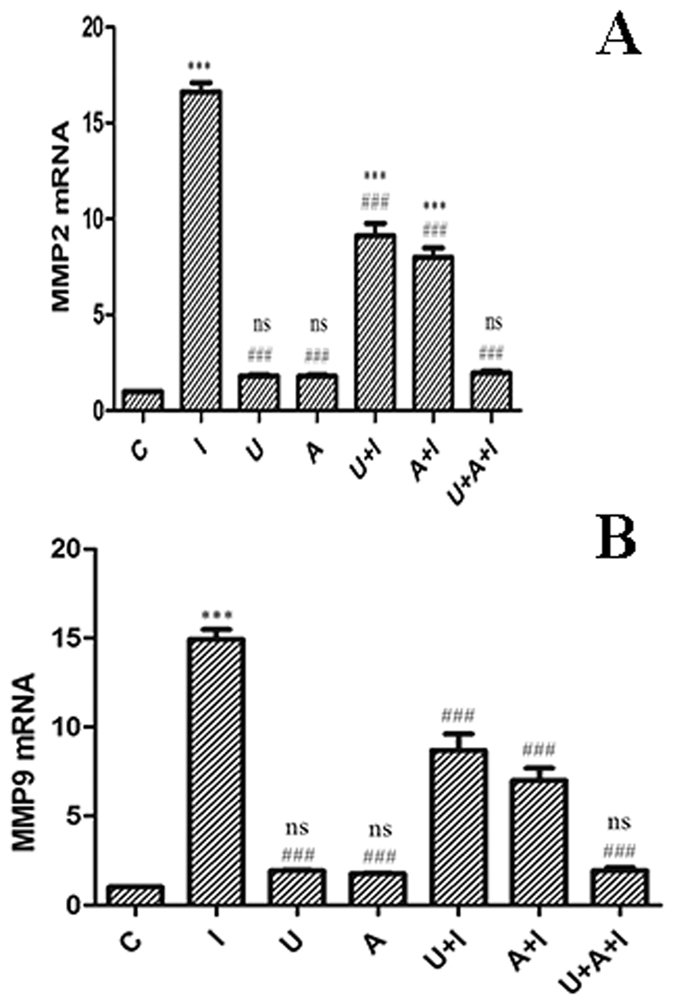
Effect of hITF on transcriptional levels of MMP-2 and MMP-9. Cells were treated with DMSO (C), hITF (I), MEK inhibitor, U0126 (U), JAK inhibitor, AG490 (A), U0126 and hITF (U + I), AG490 and hITF (A + I), both inhibitors and hITF (U + A + I) for 3 h. The levels of *MMP-2* and *MMP-9* mRNA were analyzed by real-time RT-PCR (***P < 0.001 ns no significant difference vs. control group; ^###^*P* < 0.001 vs. ITF group n = 3).

Major proteases contributing to cell migration include MMP-2 and MMP-9. To investigate whether hITF promotes cell migration by up-regulating one of these proteases, quantitative real-time PCR was performed to assess *MMP2* and *MMP9* transcription. Real-time RT-PCR analyses revealed that hITF treatment resulted in a 16–17-fold increase in *MMP2* mRNA levels and a 15-fold increase in *MMP9* transcription compared to those of the control group. Treatment with the JAK and MEK inhibitors alone resulted in an 8–9-fold decrease in *MMP2* and *MMP9* mRNA levels compared to those of the hITF-treated group, while treatment with both inhibitors decreased *MMP2* and *MMP9* mRNA levels to those of the control levels (Fig. 5).

### hITF alters E-cadherin expression

**Figure 6:**
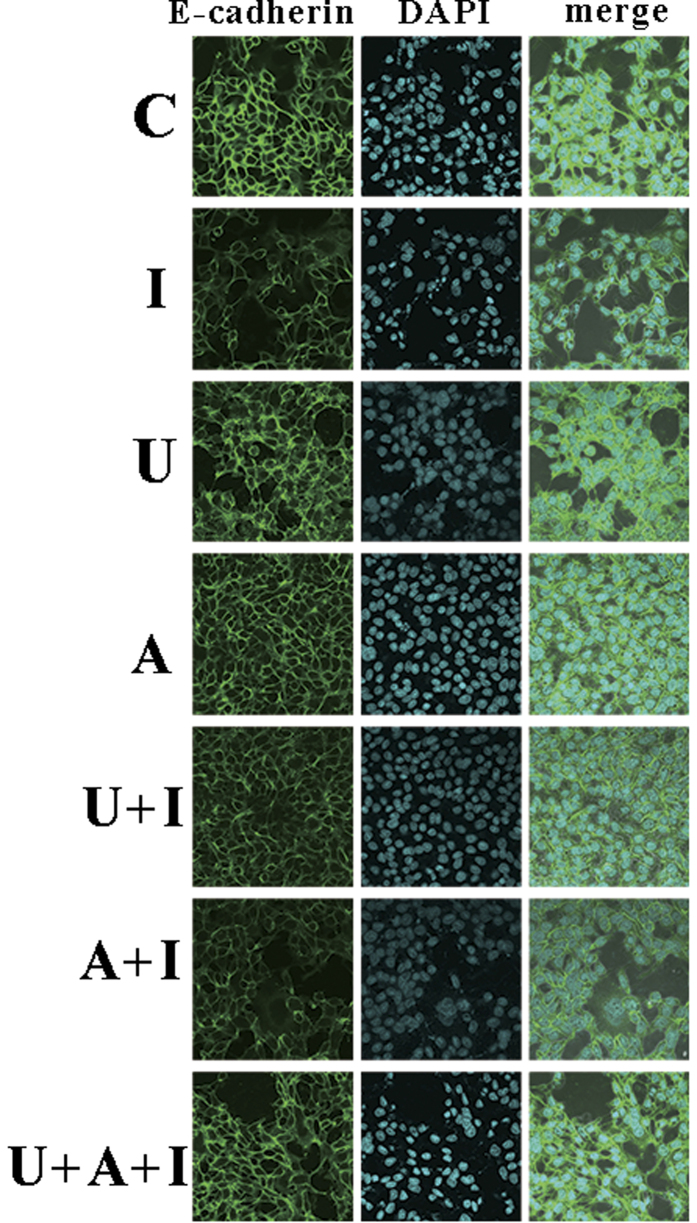
Representative immunofluorescence analysis showing E-cadherin degradation in HT-29 cells. Cells were treated with DMSO (C), indicated hITF concentrations (I), MEK inhibitor, U0126 for 2 h (U), JAK inhibitor, AG490 for 3 h (A), U0126 pretreatment and hITF (U + I), AG490 pretreatment and hITF (A + I), both inhibitors and hITF (U + A + I) for 48 h. Green: E-cadherin; blue: nucleus, 4′, 6-diamidino-2-phenylindole. The scale bar in insets represents 50 μm (original magnification: 60×).

In order to examine the effect of hITF on E-cadherin degradation, an immunofluorescence assay was performed. The results demonstrated that hITF induced a stronger reduction of E-cadherin expression compared to that of the control group. Inhibition of the ERK and JAK/STAT3 pathways rescued E-cadherin from degradation (Fig. 6).

## Discussion

hITF is a small peptide. Its main function is to alleviate gastrointestinal mucosal injuries caused by a variety of factors and to promote the repair of damaged mucosa. It plays a key role in the maintenance of the surface integrity of the intestinal mucosa and enhances healing of the gastrointestinal mucosa. Nevertheless, the mechanisms underlying these effects of ITF are unclear.

The current study is the first to provide insights into the role of the ERK and JAK/STAT3 pathways in hITF-induced cell migration. Furthermore, a molecular crosstalk was identified between the ERK and JAK/STAT3 pathways and it was involved in the effects of hITF on cell migration. Using an epithelial cell culture system, we identified bidirectional interactions between the ERK and JAK/STAT3 pathways that operate contextually within a single epithelial cell type to regulate cell migration.

We verified that hITF activated not only the ERK signaling pathway, but also the JAK/STAT3 pathway in a time dependent manner. ERK phosphorylation peaked at 1 h after hITF stimulation. Similarly, STAT3 Tyr^705^ phosphorylation peaked at 15 min after hITF treatment, but STAT3 Ser^727^ phosphorylation peaked at 1 h after treatment. This result suggests that STAT3 Ser^727^ phosphorylation might be related to the activation of the ERK pathway.

Published studies showed that cross-phosphorylation between tyrosine and serine/threonine protein kinases results in widespread crosstalks between different signaling pathways. ERK presents two phosphorylation sites, a tyrosine kinase and a serine kinase phosphorylation site^7^. The serine kinase phosphorylation site is activated by MEK, while the tyrosine kinase phosphorylation site can be activated by the JAK kinase, which belongs to the JAK-STAT3 signaling pathway. Similarly, the JAK-STAT3 signaling pathway presents two key phosphorylated sites: the Tyr^705^ phosphorylated site and Ser^727^ phosphorylated site^8^. The Tyr^705^ phosphorylated site is necessary for excitation of STAT3, which is activated by JAK and Src. The Ser^727^ is activated by several kinases such as PKC epsilon, mTOR, and ERK. Cross-phosphorylation between tyrosine and serine/threonine protein kinases may support the widespread crosstalk between ERK and JAK/STAT3 signaling pathways^9^. Subsequently, the crosstalk between the ERK and JAK/STAT3 pathways was confirmed in related research. Arredondo *et al*. used a Raf inhibitor to inhibit the Raf-MEK-ERK signaling pathway, resulting in reduced ERK activity and accidental reduced expression of STAT3 mRNA^5^. Moreover, inhibition of the JAK2/STAT3 pathway resulted in the down-regulation of ERK phosphorylation induced by IL-6 6. To verify the crosstalk mechanism between the ERK and JAK/STAT3 pathways activated by hITF, we inhibited the ERK pathway and evaluated the phosphorylation of STAT3 Tyr^705^ and Ser^727^. Conversely, we inhibited the JAK/STAT3 pathway and evaluated ERK phosphorylation. ERK inhibition decreased STAT3 Ser^727^ phosphorylation levels, but had no significant effect on phospho-STAT3 Tyr^705^. Similarly, JAK inhibition abolished the activation of the ERK pathway. This result implied that hITF activated a crosstalk between the ERK and JAK/STAT3 pathways.

While we verified the crosstalk mechanism, its effect on cell migration remained unknown. To explore the effect of this crosstalk on cell migration, a transwell assay was performed. ITF promoted cell migration and ERK or JAK inhibitor abrogated this effect. Furthermore, the number of migrating cells was lower after treatment with both the ERK and JAK inhibitors than after treatment with either inhibitor alone. This indicated that the crosstalk mechanism participated in cell migration.

It is well known that an increased expression of MMPs is important for cell migration. Among the MMPs, MMP-2 and MMP-9 are crucial enzymes in this process, degrading type I-V collagen and regulating various cell behaviors^10^. Studies showed that ITF increases the expression of MMPs to promote cell migration and the JAK/STAT3 pathway^11^,^12^. Qianqian Zheng *et al*. showed that the expression of cell–matrix molecule such as MMP-9 was enhanced by TFF3 13. Victor Y.W. Chan *et al*. found that the TFF3-induced invasiveness of Rat-2 was associated with enhanced expression of MMP-9 14. To investigate whether the crosstalk mechanism mentioned above affects *MMP2* and *MMP9* transcription, quantitative real-time PCR was performed. hITF increased *MMP2* and *MMP9* mRNA levels. Treatment with the JAK or MEK inhibitor alone could not decrease the mRNA level to the normal level, while using both inhibitors restored the normal levels. This result provided evidence that MMP2 and MMP9 are targets of ITF, promoting cell migration through the crosstalk mechanism described above.

The cell adhesion molecule, E-cadherin, is a transmembrane glycoprotein whose cytoplasmic domain is complexed with β-catenin^15^. According to the literature, several growth factors can induce the loss of E-cadherin expression and function involved in cell adhesion and migration^16^. Victor Y.W. Chan *et al*. demonstrate that the enhanced invasiveness in rat fibroblast cells by sense TFF3 transfection was associated with significant reduction in E-cadherin^14^. This study also investigated the expression of molecules involved in cell adhesion and migration. In order to examine E-cadherin degradation, an immunofluorescence assay was performed. hITF induced a strong reduction in E-cadherin expression. In addition, treatment of HT-29 cells with both the MEK and JAK inhibitors rescued E-cadherin from degradation. This finding provided convincing evidence that the crosstalk between the ERK and JAK/STAT3 pathways induced by hITF regulated the expression of the cell adhesion molecule, E-cadherin.

In conclusion, our findings argue for a third putative mechanism in the capacity of the JAK/STAT3 pathway to crosstalk with and to modulate the function of the ERK pathway and, conversely, in the capacity of ERK to crosstalk with the JAK/STAT3 signaling pathway, which are characteristics that are generally associated with cell migration induced by hITF. ERK-inhibition down-regulates STAT3 activation and vice versa. Thus, inhibition of just one of these pathways advantageously reduces the activity of the respective crosstalk partner. The cell migration was modulated by the crosstalk between the two pathways. Moreover, we confirmed that cell migration molecules, MMP2 and MMP9, and the cell adhesion molecule, E-cadherin, were targets of hITF and involved in hITF-induced cell migration. Therefore, the function of the ERK pathway during cell migration necessitates the study of additional pathways that crosstalk with the JAK/STAT3 pathway. Our study reveals a potential mechanism of the impaired intestinal reconstruction. A therapeutic strategy using these therapeutic targets might therefore exert synergistic beneficial effects on the reconstruction of damaged intestinal mucosa.

Altogether, our results suggest that hITF may exert its effects through a crosstalk between the ERK and JAK/STAT3 pathways. In particular, we show that the crosstalk mechanism induced by hITF leads to cell migration and strongly affects the expression of molecules involved in cell migration.

## Materials and Methods

### Preparation of recombinant human intestinal trefoil factor (hITF)

hITF was produced in human embryonic kidney cell line (HEK) 293 cells (purchased from ATCC, Manassas, VA, USA), which were infected with a recombinant adenovirus containing human ITF gene (Ad-hITF). The hITF gene was modified with a polyhistidine tag sequence at the N–terminal by PCR technology, His–tagged rhITF was purified to homogeneity by Ni–NTA affinity chromatography according to manufacturer’s instructions. In brief, the Ni–NTA resin slurry was washed with distilled water and equilibrated with the binding buffer containing 10 mM imidazole. The solution containing soluble proteins was loaded onto the column. Weakly bound proteins were washed from the resin by the washing buffer containing 20 mM imidazole. Bound proteins were eluted with the eluting buffer containing 250 mM imidazole.

The concentration of hITF was measured by ITF ELISA kit (Shanghai Bangyi Biotechnology Co. Ltd, Shanghai, China).

### Cell culture and treatment

Human embryonic kidney (HEK) cell line HEK293 and colon cancer cell line HT-29 were purchased from ATCC (Manassas, VA, USA). HEK293 cells were cultured in DMEM supplemented with 10% fetal bovine serum, and penicillin and streptomycin (100 U/mL) at 37 °C in 5% CO_2_. Culture medium was replaced every other day and cells were passaged every 3–4 days at a ratio of 1:3.

To generate the *in vitro* model of the human intestinal epithelium, HT-29 cells were grown in Roswell Park Memorial Institute (RPMI) 1640 supplemented with 10% fetal bovine serum (Zhejiang tianhang Biological technology stock Co. ltd, Zhejiang, China), penicillin (100 units/mL), and streptomycin (100 μg/mL) in 37 °C humidified incubator with 5% CO_2_. To determine the optimal incubation time with ITF to activate ERK and JAK/STAT3 pathways, we seeded cells on 6-well culture plates at a density of 1.2 × 10^6^ cells per well. Once 80 to 90% confluency was reached, cells were supplemented with 0.1% (v/v) fetal bovine serum to drive cell population into G0-G1 phase and incubated with ITF (60 μg/mL) for different times as indicated in the figures. In inhibition studies, cells were pretreated with the MAPK inhibitor, U0126, (10 μM) for 2 h^17^ or the JAK2 inhibitor AG490 (50 μM) for 3 h^18^ alone or followed by treatment with hITF.

### Western blot analysis

After treating HEK293 cells with Ad-ITF, cell culture supernatants were collected for examination of soluble ITF. Cell pellets were lysed with RIPA buffer and protease inhibitors, centrifuged at 12,000 rpm at 4 °C for 15 min, and the protein concentration of the lysates was quantified using the BCA method. Forty micrograms of total protein isolated from untreated or treated HT-29 cells were separated on 10% SDS-polyacrylamide gels at 80 V for 1 h and 120 V for 1.5 h. The proteins on the SDS-polyacrylamide gel were transferred to a Polyvinylidene Fluoride membrane at 350 mA at 4 °C. The membrane was then immersed in a blocking buffer and incubated with the primary antibodies overnight at 4 °C. Subsequently, the membrane was washed and incubated with the secondary antibodies conjugated with Alkaline Phosphatase at room temperature for 10 min. After washing three times with TBST for 10 min each time, proteins were detected with BCIP/NBT Color Development Substrate. The proteins were quantified and analyzed using Image J software (National Institutes of Health, Maryland, USA). β-actin served as an internal control.

### Migration assay

The migratory ability of HT-29 cells was assessed using transwell chamber (6.5 mm insert diameter, 8 μm pore size) (Corning, Corning, NY, USA), placed in 24-well culture plates. Briefly, differently treated HT-29 cells (1 × 10^5^ per well) were suspended in serum-free RPMI-1640 and were added to the top chamber of the Transwell plates and the lowerchamber were filled with 1 mL RPMI-1640 containing 10% FBS. Following 24 h of incubation at 37 °C, cells located on the upper membranes were removed with cotton swabs and the cells that migrated were fixed with 95% ethanol and stained with 0.1% crystal violet for 10 min. The cells that migrated to the lower sides of the filter were counted with an inverted fluorescence microscope. The number of migrating cells was determined by counting five random fields on each membrane.

### Total RNA extraction and Reverse Transcription

HT-29 cells were cultured in 6-well plates with or without treatment of U0126, AG490, and ITF. TRIzol (TIANGEN, Beijing, China) was used to extract total RNA according to the manufacturer’s instruction, with satisfactory quantity and quality of RNA preparations, as measured by A260 nm and A280 nm readings. First-strand cDNA synthesis was carried out with 3 μg of total RNA using Transcript RT Kit (TIANGEN, Beijing, China) according to the manufacturer’s instructions and all reactions were carried out in a Programmable Thermal Controller.

### Quantitative real-time PCR for Gene transcription

Gene transcription was measured by real time PCR (RealMasterMix SYBR Green kit, TIANGEN, Beijing, China). Each 20 μL reaction contained 2 μL of cDNA solution (produced by RT reaction described above), 0.6 μL (final concentration 300 nM) of the forward and reverse primers for the gene of interest. β-actin served as an internal control. Forward and reverse primers used were as follows:

*MMP-2*: 5′-AACTACAACTTCTTCCCTCGCAA;

5′-CAAAGGCATCATCCACTGTCTCT

*MMP-9*: 5′-CCACCCTTGTGCTCTTCCCTG;

5′-TCTGCCACCCGAGTGTAACCA

*β-actin*: 5′-GGGAAATCGTGCGTGACATTAAGG;

5′-CAGGAAGGAAGGCTGGAAGAGTG

Samples were run in biological and technical triplicate, and experiments were performed twice. The data from the quantitative real time PCR measurements were calculated using the ΔΔCt method.

### Immunofluorescence

HT-29 cells were grown on glass coverslips in 6-well plates and in the presence or absence of U0126, AG490, and hITF. The cells were briefly washed with phosphate-buffered saline (PBS) and fixed with 4% paraformaldehyde for 10 min. Subsequently, the cells were washed in PBS and permeabilized with 0.1% Triton X-100 in PBS for 10 min. After blocking with 0.1% (v/v) goat serum in PBS for 60 min at room temperature, cells were incubated with the first antibody (anti-E-cadherin antibody: dilution, 1:100; anti-β-catenin antibody: dilution, 1:100) overnight at 4 °C. After three washes in PBS, cells were incubated with FITC/TRITC-conjugated secondary antibodies goat anti-rabbit IgG for another 60 min (diluted 1:1000 in PBS). The nuclei were stained with DAPI and images were captured using a laser scanning confocal microscope.

### Statistical methods

Unless otherwise specified, all experiments were repeated three times and similar results were obtained. The results are expressed as the mean ± standard deviation. Statistical analysis was performed using one-way ANOVA; *P* < 0.05 was considered statistically significant. The data were analyzed using GraphPad Prism version 5.0 for Windows (GraphPad Prism, San Diego, CA, USA).

## Additional Information

**How to cite this article**: Le, J. *et al*. ITF promotes migration of intestinal epithelial cells through crosstalk between the ERK and JAK/STAT3 pathways. *Sci. Rep.* **6**, 33014; doi: 10.1038/srep33014 (2016).
